# Genetic structure is stronger across human-impacted habitats than among islands in the coral *Porites lobata*

**DOI:** 10.7717/peerj.8550

**Published:** 2020-02-18

**Authors:** Kaho H. Tisthammer, Zac H. Forsman, Robert J. Toonen, Robert H. Richmond

**Affiliations:** 1 Kewalo Marine Laboratory, University of Hawaii at Manoa, Honolulu, HI, United States of America; 2 Department of Biology, San Francisco State University, San Francisco, CA, United States of America; 3 Hawaii Institute of Marine Biology, University of Hawaii at Manoa, Kaneohe, HI, United States of America

**Keywords:** Coral reefs, Anthropogenic impacts, Population genetics, Hawaii, Lobe coral, Isolation by distance, Isolation by environment, Local adaptation, Gene flow

## Abstract

We examined genetic structure in the lobe coral *Porites lobata* among pairs of highly variable and high-stress nearshore sites and adjacent less variable and less impacted offshore sites on the islands of Oahu and Maui, Hawaii. Using an analysis of molecular variance framework, we tested whether populations were more structured by geographic distance or environmental extremes. The genetic patterns we observed followed isolation by environment, where nearshore and adjacent offshore populations showed significant genetic structure at both locations (AMOVA *F*_*ST*_ = 0.04∼0.19, *P* < 0.001), but no significant isolation by distance between islands. Strikingly, corals from the two nearshore sites with higher levels of environmental stressors on different islands over 100 km apart with similar environmentally stressful conditions were genetically closer (*F_ST_* = 0.0, *P* = 0.73) than those within a single location less than 2 km apart (*F_ST_* = 0.04∼0.08, *P* < 0.01). In contrast, a third site with a less impacted nearshore site (i.e., less pronounced environmental gradient) showed no significant structure from the offshore comparison. Our results show much stronger support for environment than distance separating these populations. Our finding suggests that ecological boundaries from human impacts may play a role in forming genetic structure in the coastal environment, and that genetic divergence in the absence of geographical barriers to gene flow might be explained by selective pressure across contrasting habitats.

## Introduction

Coral reefs are centers of marine biodiversity and productivity that provide a variety of ecosystem services of substantial cultural and economic value to humankind, yet coral reefs worldwide are under serious threat as a result of human activities (Hughes et al., 2010; Graham, 2014). Average global coral cover has declined dramatically in the past 100 years due to a range of impacts such as sedimentation, pollution, overfishing, disease outbreaks and climate change (Hughes et al., 2010; Richmond & Wolanski, 2011; Graham, 2014).

Such effects are particularly pronounced in nearshore marine habitats, which are increasingly exposed to reduced water quality due to human activities (Wenger et al., 2015). Recent rapid coastal development, along with coastal industrial and recreational activities, have resulted in introducing sediments, nutrients and a variety of chemical pollutants to the nearshore environments (Smith et al., 2008b; Van Dam et al., 2011; Wenger et al., 2015). These local stressors often create a steep environmental gradient of water quality from nearshore toward offshore areas, and ‘signs of coral health impairment’ are usually detected along with the gradient (e.g., Smith et al., 2008b; Thompson et al., 2014; Ennis et al., 2016). Additionally, nearshore marine habitats are naturally exposed to higher fluctuations in temperature, pH and other environmental variables, creating contrasting environmental conditions relative to more stable offshore environments (Gorospe & Karl, 2011; Guadayol et al., 2014). Some corals, however, continue to thrive in such nearshore ‘suboptimal’ habitats (Morgan et al., 2016), indicating that these individuals can withstand such stressors.

What impact does an ecological landscape with such a strong gradient have on the genetics of the organisms? ‘Isolation by distance’ (IBD, Wright, 1943) predicts that the degree of genetic differentiation increases with geographic distance due primarily to dispersal limits (Slatkin, 1993; Selkoe et al., 2016). Isolation by distance is a neutral process in which dispersal limits gene flow and the scale over which genetic structure accumulates. ‘Isolation by environment’ (IBE, Wang & Bradburd, 2014) describes a pattern in which genetic differentiation increases with environmental differences, independent of geographic distance. Isolation by environment is a process that emphasizes the role of environmental heterogeneity and ecology in forming genetic structure, likely because of natural selection (Orsini et al., 2013; Wang & Bradburd, 2014). Isolation by environment can be generated by different processes, including natural selection, sexual selection, reduced hybrid fitness, and biased dispersal; examples of the terms describing a specific case of IBE include ‘isolation by adaptation’ (IBA, Nosil, Funk & Ortiz-Barrientos, 2009), ‘isolation by colonization’ (IBC, De Meester et al., 2002), and ‘isolation by resistance’ (IBR, McRae & Beier, 2007). IBA and IBC emphasize the role of selection in forming genetic structure, and IBR describes correlation of genetic distance and resistance distance (i.e., friction to dispersal) (McRae & Beier, 2007). IBE along with related terms result in a pattern where genetic distance increases as ecological distance increases, but not with geographic distance for most loci (Orsini et al., 2013; Wang & Bradburd, 2014). Theoretically, IBA, IBC and other processes will result in different distributions of genetic variation across landscapes (Orsini et al., 2013), though in reality, multiple processes almost always contribute to structuring genetic variation, and pinpointing the possible underlying processes may be difficult (Selkoe et al., 2016). For coastal marine ecosystems, often the distances between the impacted nearshore and un-impacted offshore sites are relatively small with no apparent dispersal barrier between adjacent sites for broadcast spawning species with pelagic larval development, providing an excellent opportunity to study IBE.

Several studies have shown coral genetic divergence occurring along ecological gradients. For example, Carlon and Budd (Carlon & Budd, 2002) described a pair of incipient species in the coral *Favia fragum* associated with strong ecological gradients. The two types are largely restricted to alternate seagrass and adjacent coral reef habitats, but retain phenotypic distinction in a narrow zone of ecological overlap (Carlon & Budd, 2002). Subsequent work showed that the morphologies were heritable, and selection appeared to limit gene flow between the ecomorphs (Carlon et al., 2011). Carlon et al. (2011) postulated that divergent selection for “Tall” and “Short” ecomorphs of these inbred and brooding corals was driving the diversification of this coral via an ecological model of speciation (*sensu* Rundle & Nosil, 2005). Genetic divergence across nearshore and costal headland habitats has also been observed for a broadcast spawning coral Porites lobata in American Samoa (Barshis et al., 2010), and for brooding corals *Seriatopora hystrix* in Australia (Bongaerts et al., 2010) and Porites astreoides in the Florida Keys (Kenkel et al., 2013). Additionally, Gorospe & Karl (2015) found a significant genetic cline in *Pocillopora damicornis* along a depth gradient across a 40 m diameter patch reef in Hawaii.

*Porites lobata* (Dana, 1846), the study species, occurs over a wide geographic range in the tropical Pacific Ocean (Veron, 2000), and several studies have documented a pattern of isolation by distance across archipelagic (Polato et al., 2010) or broader scales (Baums et al., 2012), with little evidence of restricted gene flow among geographically proximate reefs at inter-island distances (but see Barshis et al., 2010). This massive coral is also known for its robustness; for example, *P. lobata* shows a high tolerance for sedimentation (Stafford-Smith, 1993) and bleaching (Levas et al., 2013), and a colony can recover from partial mortality due to tissues residing deep within the perforate skeleton, a phenomenon referred to as the ‘Phoenix effect’ (Roff et al., 2014). *Porites lobata* is one of the most dominant scleractinian coral species in Hawaii (Franklin, Jokiel & Donahue, 2013). Additionally, *P. lobata* shows high fidelity to a specific endo-symbiont, *Symbiodinium* Clade C15, which allows us to focus on responses of the host coral to environmental differences (LaJeunesse et al., 2004; Smith et al., 2008a; Barshis et al., 2010; Fabina et al., 2012).

Skeletal morphological differences for *P. lobata* appear to tell a similar story as for *F. fragum* (Forsman et al., 2015; Tisthammer & Richmond, 2018), though environmental gradients have not yet been tested in population genetic studies of this species in Hawaii. A growing number of studies indicate that IBE may be more ubiquitous in the sea than previously assumed. Therefore, we pose the question of whether there is reason to believe non-IBD patterns might be observed in contrasting habitats in Hawaii. In order to test our hypothesis, we implement a non-traditional population genetics sampling design that allows us to consider whether geography *vs.* environmental gradients explain overall patterns of genetic differentiation.

Maunalua Bay, Hawaii, Oahu, was selected as a study site due to the existence of a strong environmental gradient; large-scale urbanization in adjacent watersheds has caused severe deterioration in the health and extent of its nearshore coral reefs over the last century (Wolanski, Martinez & Richmond, 2009). Corals that survive in these affected nearshore areas are under chronic stress, and a previous survey showed significantly different cellular stress responses of individual colonies along the environmental gradient of pollutants and sedimentation from the inner bay toward offshore (Wolanski, Martinez & Richmond, 2009; Richmond, 2009; Storlazzi et al., 2010). At the nearshore site of Maunalua Bay, the suspended sediment concentration periodically exceeds several hundred mg/L, and the run-off water introduces toxicants such as benzo[a]pyrene, benzo[k]fluoranthene, phenanthrene and alpha-chlordane (Wolanski, Martinez & Richmond, 2009; Richmond, 2009; Storlazzi et al., 2010). The temperature, salinity and turbidity likewise all show higher ranges of values and fluctuations at the nearshore site than the offshore site (Storlazzi et al., 2010). The distance between the studied offshore and nearshore sites was less than 2 km across the extent of this gradient, and water movement in the areas suggests no dispersal barrier between the sites (Presto et al., 2012).

Wahikuli, West Maui, was selected as another study site due to the existence of a sharp environmental gradient that also runs from nearshore to offshore. Although less well characterized than Oahu, the coral reefs off West Maui have a similar gradient of human impacts and experienced a dramatic decline in their coral cover from land-based sources of anthropogenic stressors over the last several decades (Rodgers et al., 2015). Substantial deterioration in the health of West Maui’s coral reefs has lead Wahikuli and Honokōwai watersheds of West Maui to be designated as priority sites for conservation and management by the United States Coral Reef Task Force (USCRTF) and the State of Hawaii (Williams et al., 2014). The Wahikuli study site is directly exposed to terrestrial run-off, due to its topography and current patterns (Storlazzi et al., 2006), causing high turbidity especially after heavy rains. Despite their proximity, the nearshore area at Wahikuli has markedly different water quality than offshore reefs roughly 300 m away (Fig. S2). In contrast, the nearshore area at the Honokōwai site is less affected by runoff, because it does not receive any direct stream discharge, resulting in consistently lower turbidity than the Wahikuli nearshore site (Vargas-Angel, 2017).

**Figure 1:**
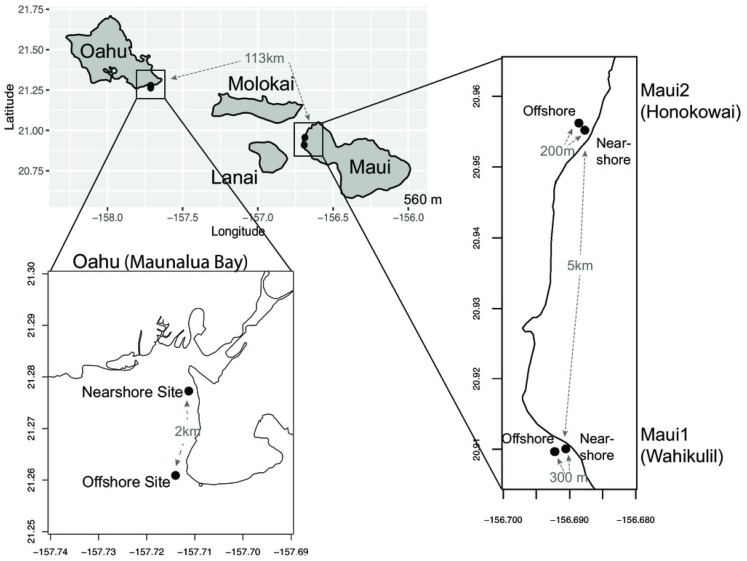
Maps of sampling locations. The Main Hawaiian Islands from which samples of *Porites lobata* were sampled. Paired sites at each location are designated as Nearshore (N) or Offshore (O) and geographic distances between the sampling sites are shown in gray.

*Porites lobata* populations from Maunalua Bay, Oahu (hereafter Oahu) and Wahikuli, Maui (hereafter Maui1) represent two locations with a strong environmental gradient, whereas Honokōwai, Maui (hereafter Maui2) represents a similar paired nearshore-offshore comparison site, but without a strong environmental gradient (Fig. 1). Nearshore and offshore sites at Oahu and Maui1 sites are both characterized by highly contrasting environments in proximity, with dramatic differences in water quality and sedimentation loads from anthropogenic impacts. We undertook a genetic analysis of *P. lobata* across these sites to explore the possibility of isolation by environment. By comparing corals collected from heavily impacted nearshore environments to nearby congeners from more oceanic conditions, we sought to distinguish the roles played by ecology and anthropogenic impacts to the environment on the genetics of coral populations, in contrast to geographical distance limiting dispersal among similar habitats on adjacent islands. We predicted that the genetic structure of coral populations from areas with a strong environmental gradient would follow IBE, rather than IBD. At each location, we assessed the degrees of genetic differentiation and genetic diversity of *P. lobata* between adjacent strongly anthropogenically impacted ‘higher-stress’ nearshore and ‘lower-stress’ offshore sites, and compared them within and between locations to understand the effects of habitat types, anthropogenic impacts, and geographical distance on the genetic structure of reef building corals.

## Materials & Methods

### Species identification

Due to its high morphological variation, the genus *Porites* is notorious for its difficulties in distinguishing between its species (e.g., Veron, 1995; Veron, 2000; Forsman et al., 2009; Forsman et al., 2015). Genetic delineation of some *Porites*, including *P. lobata*, has been challenging due to cryptic species and polymorphic or hybrid species complexes (e.g., Veron & Pichon, 1982; Forsman et al., 2009). Although *Porites* corallites are small, irregular and can be highly variable, micro-skeletal (corallite) structures have been proposed to be important for species identification, therefore, we examined the corallites of all collected samples to confirm our taxonomic identifications (Veron & Pichon, 1982; Veron, 2000) (Fig. S3). In Hawaii, the only *Porites* species with a similar colony morphology to *P. lobata* is *Porites evermanni* (there are no records of *Porites lutea* in Hawaii, although Fenner (2005) synonymized *P. evermanni* and *P. lutea*, they represent two distinct genetic clades (Forsman et al., 2009). *P. evermanni* is genetically distinct from *P. lobata* (Forsman et al., 2009), Clade V), and *P. lutea* has a distinct corallite skeletal morphology, compared to *P. lobata* (Veron, 2000).

### Coral sampling

Small fragments (1 cm^2^) of *P. lobata* tissue samples were collected from live colonies between February 2013 to May 2017 at the following sampling sites in Hawaii; (a) ‘Oahu’—nearshore (*n* = 22) and offshore (*n* = 21) sites at Maunalua Bay, Oahu (21.261∼21.278°N, 157.711°W), (b) ‘Maui1’—nearshore (*n* = 21) and offshore (*n* = 23) sites off the Hanakao’o Beach Park, West Maui (Wahikuli, 20.95°N, 156.68°W), and (c) Maui2—nearshore (*n* = 23), and offshore (*n* = 20) sites off the Honokōwai Beach Park, West Maui (Honokōwai, 20.90°N, 156.69°W) (Fig. 1, Fig. S1). Samples were taken from coral colonies at least two meters apart at each site, and after sampling, each coral colony was photographed and tagged to avoid resampling of the same colony. The collected tissue samples were either flash frozen in liquid nitrogen on shore and subsequently stored at -80, preserved in DMSO buffer Gaither et al., 2011) , or stored in 100% ethanol. Genomic DNA was extracted from each coral tissue sample using the Qiagen® DNeasy Blood & Tissue Kit. Coral samples were collected under the State of Hawaii Division of Aquatic Resources, Special Activity Permit 2013-26, 2014-64, 2015-06, and 2017-16.

### PCR

For the samples from Oahu, the following three regions of coral host DNA were PCR-amplified: (1) ∼400 bp coral mitochondrial putative control region (CR) with primers CRf and CO3r (Vollmer & Palumbi, 2002), (2) ∼700 bp coral nuclear ITS1-5.8S-ITS2 region (ITS) with primers ITSZ1 and ITSZ2 (Forsman et al., 2009), and (3) ∼1,500 bp coral nuclear histone region spanning H2A to H4 (H2) with novel primers zH2AH4f (5′-GTGTACTTGGCTGCYGTRCT-3′) and zH4Fr (5′-GACAACCGAGAATGTCCGGT-3′). H2 was developed to create a genetic marker that allow direct sequencing of post PCR products to efficiently assess small-scale population genetic structure, because (1) the mitochondrial genome of *P. lobata* exhibits very little sequence variability (<0.02% polymorphic sites (Tisthammer et al., 2016)) due to its extremely slow evolutionary rate (Shearer et al., 2002), and (2) even though high polymorphism in ITS is a desirable trait, sequencing of ITS requires time-consuming cloning, and analyzing the multi-copy gene poses analytical challenges, as it deviates from a standard diploid model. H2 consists of partial coding region of H2A, approximately 1000 bp introns and part coding region of H4. After successful development of the H2 marker, Maui populations were analyzed with only H2 for the above reasons (see File S1 for more details).

H2 was amplified under the following conditions: 96 °C for 2 min (one cycle), followed by 34 cycles consisting of 96 °C for 20 s, 58.5 °C for 20 s, and 72 °C for 90 s, and a final extension at 72 °C for 5 min. H2 amplifications (25 µl) consisted of 0.5 µl of DNA template, 0.2 µl of GoTaq® DNA Polymerase (Promega, Madison, WI), 5 µl of GoTaq® Reaction Buffer, 1.6 µl of 50 mM MgCl_2_, 2 µl of 10 mM dNTPmix, 1.6 µl of each 10 mM primer, and nuclease-free water to volume. For samples with multiple bands, approximately 1500-bp PCR products were extracted from agarose gels after electrophoresis and purified using the UltraClean® 15 DNA Purification Kit (MO BIO Laboratories, Carlsbad, CA) according to the manufacturer’s instruction. The rest of the PCR products were purified with UltraClean® PCR Clean-Up Kit (MO BIO Laboratories) and sequenced directly in both directions on the ABI 3730xl DNA Analyzer. Clone libraries were created for each amplified ITS region using the pGEM®-Easy Vector System (Promega). Positive inserts were verified by PCR using SP6 and T7 primers, and plasmids (2–5 per library) were treated with UltraClean® 6 Minute Mini Plasmid Prep Kit (MO BIO Laboratories) and sequenced on an ABI-3130XL Genetic Analyzer sequencer. For Maui samples, H2 was amplified and sequenced using the same method as described above.

### Sequence analyses

Resulting DNA sequences were aligned using Geneious® 6.1.8 (Biomatters Ltd., Auckland, New Zealand). Polymorphic sites within H2 regions were identified using Geneious® (Find Heterozygotes option) and confirmed by eye. Middle sections, as well as both ends of H2 were then trimmed to 1,352 bp (for Oahu sequences) or 1,221 bp (for combined Oahu and Maui analysis) to minimize missing nucleotides among samples. H2 was phased using the program PHASE 2.1 (Stephens, Smith & Donnelly, 2001) and SeqPHASE (Flot, 2010). The analysis of molecular variance and other population genetic statistics were estimated in Arlequin 3.5 (Excoffier & Lischer, 2010) and TCS 1.21 (Clement, Posada & Crandall, 2000). The global AMOVA with a weighted average over loci with permutation tests was used as implemented in Arlequin 3.5, using pairwise difference as a distance computation method. For H2, both phased and non-phased sequences were run with AMOVA, which produced the same statistical results, and therefore only the results from the phased sequences are presented here. Up to five coral ITS sequences were successfully cloned and sequenced per colony, and the entire data set was used for calculation of population statistics, treating each cloned sequence as a haplotype. Attempts have been made to conduct genetic analysis using ITS by (a) treating each sequence as a haplotype (inclusivity), (b) making a consensus sequence per individual (consensus by plurality), or (c) using a hierarchal PERMANOVA (Barshis et al., 2010). In this study, we ran AMOVA using ITS by both (a) and (b) methods, which produced the same statistical outcome, and hence, the results from inclusivity (a) are presented in this paper. To address the unequal sample sizes (28 vs 44) between the sites in Maunalua Bay, the analysis was repeated after resampling to the equal sample size (28) for 10 times. All DNA sequences were inspected for the possibility of multi-sampled individuals, and all sampled colonies were considered as separate individuals (genets) since no two individuals from a single site shared the same haplotypes (H2). Mantel’s test for isolation by distance was run on the samples in R (R Core Team, 2014) using pairwise genetic distance with 5000 bootstrap permutations. Rarefaction analysis was conducted in Analytic Rarefaction 2.1.1 (Holland, 2009). The network analysis was conducted in SplitsTree v.4.14.2, using UncorrectedP as a distance method, NeighborNet as a network computing method.

## Results

### Nearshore vs. offshore comparison of genetic structure and diversity of *P. lobata* populations

### Oahu (Maunalua Bay)

**Figure 2:**
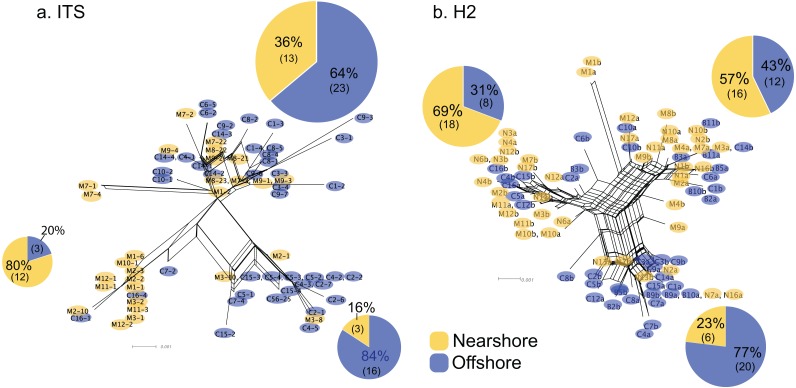
Allele networks for *Porites lobata* sampled from Oahu (Maunalua Bay). NeighborNet phylogenetic networks generated by SplitsTree, based on (A) ITS and (B) H2 color-coded by Nearshore (yellow) and Offshore (blue) sampling locations as shown in Fig. 1. Pie charts represent the proportion of sequences within each cluster.

**Figure 3:**
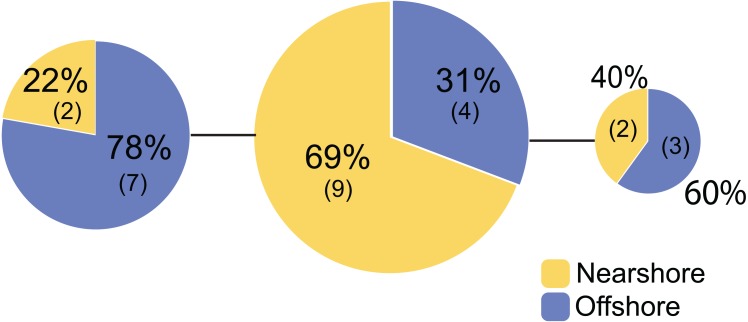
Mitochondrial haplotype network for *Porites lobata* sampled from Oahu (Maunalua Bay). Haplotype network based on mitochondrial putative control region (CR) color-coded by Nearshore and Offshore sampling locations as shown in Fig. 1.

For Oahu *P. lobata* populations, the degree of genetic differentiation was estimated using analysis of molecular variance (AMOVA (Excoffier & Lischer, 2010)) between the nearshore and offshore sites using three genetic markers; CR, ITS, and novel H2 developed for this study. The AMOVA results for both nuclear makers revealed clear genetic differentiation between the two sites (ITS, *F*_*ST*_ = 0.19, *P* < 0.001; H2, *F*_*ST*_ = 0.072, *P* < 0.001) (Table 1). The mitochondrial marker (CR) did not detect significant differentiation (*F*_*ST*_ = 0.086, *P* = 0.15), which was not surprising due to its extremely low variability in corals and cnidarians in general (Shearer et al., 2002). The numbers of shared haplotypes (alleles) between the nearshore and offshore Oahu populations were also low; out of 37 ITS haplotypes identified from the 70 total sequences, only three (8.1%) were shared between the sites. For H2, there were 54 unique haplotypes out of 86 total phased sequences, and only 5 sequences (9.3%) were shared between the sites (Table 2). The pattern of genetic structure was visualized using network analysis, which revealed sequences clustering into three major groups in both ITS and H2 markers, which consisted of one cluster dominated by the nearshore individuals, the second one dominated by the offshore individuals, and the last group with approximately mixed origins (Fig. 2). For CR, three haplotypes were identified from 27 sequences, all of which were present at both sites. Interestingly, the most common haplotype was the most dominant one at the nearshore site, while the second common haplotype was the dominant haplotype at the offshore site, though the AMOVA, as well as the exact test results were not significant (*P* = 0.15 and 0.08 respectively) (Fig. 3).

**Table 1:** Analysis of Molecular Variance (AMOVA) for *Porites lobata* collected from Oahu (Maunalua Bay).

	Source of Variation	Variance components	% Variance	*F*_*ST*_
ITS	Between populations	2.27	19.18	0.19^***^
(*n* = 70)	Within populations	9.56	80.82	
H2	Between populations	0.29	7.15	0.072^***^
(*n* = 43)	Within populations	1.30	31.88	
	Within individuals	2.49	60.96	
CR	Between populations	0.034	8.49	0.086 (*P* = 0.15)
(*n* = 20)	Within populations	0.370	91.5	

**Notes.**

Populations here refer simply to individuals sampled within the same sampling location, with sample size in parentheses below the marker. % Variance refers to the proportion of genetic variation explained by each comparison, and bold numbers with *** are significant at *P* < 0.001.

**Table 2:** Population genetic statistics of *Porites lobata* from Oahu (Maunalua Bay).

Sites	ITS (707 bp)									
	*n*	A	pA	poly	D_A_	D_P_	i	*π*	*θπ*	*θ*s
Oahu Nearshore	28	13 (46%)	10 (36%)	45 (6.4%)	1.0 ± 0.0095	0.259 ± 0.182	31	0.0167 ± 0.009	11.64 ± 5.44	3.60 ± 1.45
Oahu Offshore	42	27 (64%)	24 (57%)	70 (10%)	1.0 ± 0.0052	0.343 ± 0.192	50	0.0340 ± 0.017	24.03 ± 10.78	6.04 ± 2.06
Oahu Offshore^*^	(28)	21.7 (78%)	19.3 (69%)	65.2 (9.2%)			48.5	0.0337 ± 0.017	23.7 ± 11.96	5.37 ± 2.00

	H2 (1,352 bp)										
	*n*	A	pA	poly	Ho	He (D_A_)	D_P_	hom	*π*	*θπ*	*θ*s
Oahu Nearshore	22 (44)	28 (64%)	23 (52%)	27 (2.0%)	0.773	0.965	0.120 ± 0.151	5 (23%)	0.00553 ± 0.003	7.483 ± 3.96	6.207 ± 2.09
Oahu Offshore	21 (42)	31 (74%)	26 (62%)	27 (2.0%)	0.810	0.977	0.162 ± 0.179	4 (19%)	0.00558 ± 0.003	7.554 ± 4.00	6.275 ± 2.12

	CR (366 bp)								
	*n*	A	pA	poly	D_A_	D_P_	*π*	*θπ*	*θ*s
Oahu Nearshore	13	3 (23%)	0 (0%)	2 (0.5%)	1.0 ± 0.0302	0.282 ± 0.000	0.00154 ± 0.0015	0.5641 ± 0.551	0.6445 ± 0.485
Oahu Offshore	14	3 (21%)	0 (0%)	2 (0.5%)	0.45 ± 0.0270	0.451 ± 0.124	0.0056 ± 0.003	0.9011 ± 0.747	0.6289 ± 0.474

**Notes.**

Sample size (*n*, for H2, the number in parentheses represents the number of phased sequences), number of haplotypes (A), number of private haplotypes (pA), number of polymorphic sites (poly), mean overall gene diversity (D_A_ ± SD), mean gene diversity for polymorphic sites only (D_P_ ± SD), observed heterozygosity (H_O_), expected heterozygosity (He), number of indels (i), number of homozygous individuals (hom), nucleotide diversity (*π* ± SD), theta estimator 1 (*θπ*: expected heterozygosity at a nucleotide position estimated from the mean *π*), theta estimator 2 (Watterson estimator, *θ*s).

* Standardized values to the minimum sample size of 28.

**Figure 4:**
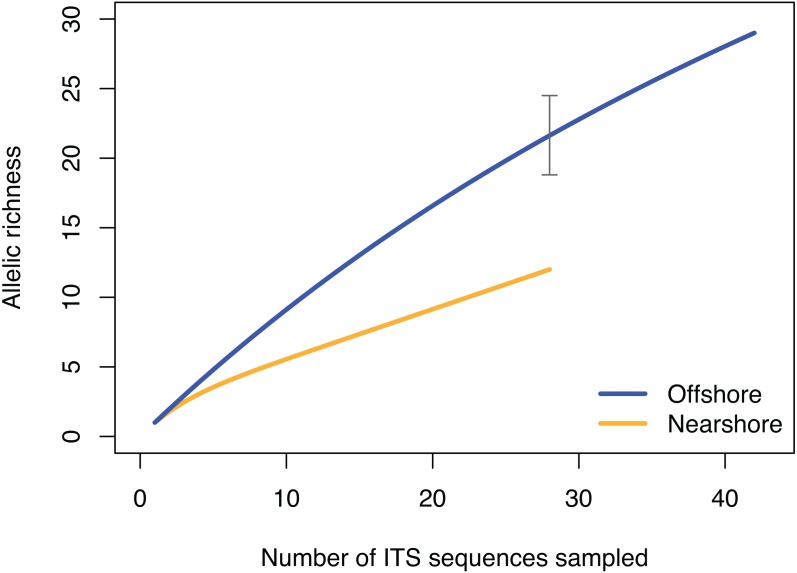
Rarefaction curve of allelic richness of ITS sequences from *Porites lobata* sampled from nearshore and offshore populations on O’ahu (Maunalua Bay). Allelic richness from individuals collected at Nearshore and Offshore sampling sites of Oahu as shown in Fig. 1. The gray bar indicates the 95% confidence interval of the allelic richness estimation for the offshore population at equivalent sample size of *n* = 28.

The pattern of genetic diversity also differed between the nearshore and offshore populations. The degree of genetic diversity was higher at the offshore site; percent private alleles (pA), percent polymorphic sites (poly), and nucleotide diversity level (*π*) were almost twice as high in the offshore population as in the nearshore one based on ITS (Table 2). Standardizing sample size by random resampling confirmed that this was not an artifact of a larger sample size of the offshore population (Table 2). Rarefaction analysis of ITS sequences also confirmed that allelic richness of the offshore population (Richness = 21.6 ± 2.1 at *n* = 28, 95% CI [18.8–24.5]) was clearly higher than that of the nearshore population (Richness = 13) (Fig. 4). The level of genetic diversity in H2 also appeared slightly higher in the offshore populations; the number of haplotypes (A), the number of private allele (pA), the heterozygosity level (H_O_), the number of heterozygous individuals, and mean gene diversity (D_A_, D_P_) all had marginally higher values in the offshore samples, though the differences were small (Table 2). In both nuclear markers, *θπ* (the expected heterozygosity estimated from the average nucleotide diversity) was higher than *θ*s (the theta estimated from the number of segregating sites).

### Maui

At the two study locations on the island of Maui, patterns of genetic structure of *P. lobata* populations between the nearshore and offshores sites were analyzed using the novel H2 marker (see Material and Methods). At Maui1, where a strong environmental gradient exists, significant genetic differentiation was detected (*F*_*ST*_ = 0.0415, *P* = 0.0308), but the level of genetic diversity was comparable between the two sites (Table 3). At Maui2, which had much less contrasting environmental conditions between the nearshore and offshore sites, the AMOVA results found no significant genetic differentiation between the nearshore and offshore sites (*F*_*ST*_ = 0.0019, *P* = 0.991). The level of genetic diversity at Maui2 appeared slightly higher in the nearshore population, which had higher numbers of haplotypes (A), polymorphic sites (poly), and heterozygous individuals (Table 3). The theta estimators of Maui2 also showed a different pattern from the Oahu and Maui1 populations, with higher values of *θ*s than those of *θπ* at both nearshore and offshore sites.

**Table 3:** Population genetic statistics of *Porites lobata* from Maui1 and Maui2 sites.

	H2 (1,221 bp)									
	*n*	A	pA	poly	hom	Ho	He	*π*	*θπ*	*θ*s
Maui1 Nearshore	18 (36)	31 (86%)	15 (83%)	27 (2.2%)	3 (17%)	0.833	0.987	0.00596 ± 0.0032	7.271 ± 3.87	6.511 ± 2.25
Maui1 Offshore	22 (44)	35 (80%)	18 (82%)	31 (2.5%)	4 (18%)	0.818	0.986	0.00590 ± 0.0031	7.209 ± 3.82	7.126 ± 2.34
Maui2 Nearshore	23 (46)	38 (86%)	43 (93%)	65 (5.3%)	3 (13%)	0.870	0.988	0.00614 ± 0.0035	7.494 ± 3.96	14.790 ± 4.48
Maui2 Offshore	20 (40)	30 (75%)	37 (93%)	33 (2.7%)	4 (20%)	0.800	0.967	0.00481 ± 0.0026	5.878 ± 3.19	7.758 ± 2.57

**Notes.**

Based on 1,319 bp of H2 sequence data. Sample size (*n*, represents the number of phased sequences), number of haplotypes (A), number of private haplotypes (pA), number of polymorphic sites (poly), mean overall gene diversity (D_A_ ± SD), mean gene diversity for polymorphic sites only (D_P_ ± SD), observed heterozygosity (H_O_), expected heterozygosity (He), number of indels (i), number of homozygous individuals (hom), nucleotide diversity (*π* ± SD), theta estimator 1 (*θπ*: expected heterozygosity at a nucleotide position estimated from the mean *π*), theta estimator 2 (Watterson estimator, *θ*s). * Standardized values to the minimum sample size of 28.

### Oahu *vs.* Maui

Inter-island genetic structure, as well as comparison of nearshore and offshore populations were conducted using H2 marker. The hierarchical AMOVA did not detect significant structure between the Oahu and Maui populations (*F*_*CT*_ = 0.007, *P* = 0.27), but the two Maui locations showed significant differentiation (*F*_*SC*_ = 0.063, *P* = 0.000) based on H2 (Table S2A). The patterns of genetic diversity suggested an overall lower variability in the Oahu population; the numbers of haplotypes (A), polymorphic sites (poly), and heterozygous individuals were all smaller on Oahu, although nucleotide diversity (*π*) levels were relatively similar between Oahu and Maui (Table 4).

**Table 4:** Population genetic statistics of *Porites lobata* from the islands of Oahu and Maui.

	H2 (1,221 bp)									
	*n*	A	pA	poly	hom	Ho	He	*π*	*θπ*	*θ*s
Maui1 (pooled)	40 (80)	63 (79%)	76 (95%)	38 (3.1%)	7 (15.9%)	0.825	0.986	0.0060 ± 0.0032	7.385 ± 3.87	7.672 ± 2.26
Maui2 (pooled)	43 (86)	68 (79%)	82 (95%)	82 (6.7%)	7 (16.2%)	0.837	0.983	0.00571 ± 0.0030	6.972 ± 3.67	16.316 ± 4.38
Maui (pooled)	83 (166)	126 (76%)	159 (81.5%)	96 (7.9%)	14 (16.1%)	0.831	0.987	0.00604 ± 0.0031	7.380 ± 3.84	16.355 ± 3.96
Oahu (pooled)	43 (86)	54 (62.7%)	47 (87%)	35 (2.9%)	9 (20.9%)	0.791	0.974	0.00643 ± 0.0033	7.844 ± 4.08	6.964 ± 2.06

**Notes.**

Based on 1,221 bp of H2 sequence data. Sample size (*n*, represents the number of phased sequences), number of haplotypes (A), number of private haplotypes (pA), number of polymorphic sites (poly), mean overall gene diversity (D_A_ ± SD), mean gene diversity for polymorphic sites only (D_P_ ± SD), observed heterozygosity (H_O_), expected heterozygosity (He), number of indels (i), number of homozygous individuals (hom), nucleotide diversity (*π* ± SD), theta estimator 1 (*θπ*: expected heterozygosity at a nucleotide position estimated from the mean *π*), theta estimator 2 (Watterson estimator, *θ*s). * Standardized values to the minimum sample size of 28.

Pairwise *F*_*ST*_ comparisons between all combinations revealed that the nearshore populations from Oahu and Maui1 with a high level of environmental stress were genetically closer to each other than to their respective, nearby offshore populations, and similarly the offshore populations from Oahu and Maui1 were genetically closer to each other than to their respective nearshore populations (Table 5, Oahu and Maui1 populations). Assessing by habitat types, the nearshore and offshore populations of Oahu and Maui1 also exhibited significant genetic differentiation (*F*_*ST*_ = 0.065, *P* = 0.000, Table S2B). The results also revealed that Maui2 corals, which showed no significant structure between the nearshore and offshore sites, turned out to be rather genetically unique compared to the rest of the populations. However, the *F*_*ST*_ values indicated that the Maui2 nearshore population was genetically closer to other offshore populations (*F*_*ST*_ = 0.027–0.035) than to other nearshore populations of Oahu and Maui1 (*F*_*ST*_ = 0.14–0.18), suggesting collectively that Maui2 corals at both sites were genetically closer to the offshore populations (Table 5).

**Table 5:** Pairwise *F*_*ST*_ values for *Porites lobata* sampled from Oahu and Maui.

	Oahu N	Oahu O	Maui1 N	Maui1 O	Maui2 N	Maui2 O
Oahu N	–	0.000	0.324	0.000	0.000	0.000
Oahu O	0.079^***^	–	0.008	0.666	0.012	0.046
Maui1 N	0.001	0.055^**^	–	0.004	0.000	0.000
Maui1 O	0.077^***^	−0.007	0.052^**^	–	0.015	0.014
Maui2 N	0.177^***^	0.035^*^	0.139^***^	0.027^*^	–	0.072
Maui2 O	0.205^***^	0.026^*^	0.170^***^	0.030^*^	0.013	–

**Notes.**

Pairwise *F*_*ST*_ values were estimated using AMOVA in Arlequin with 5,000 permutations. Below diagonal = *F*_*ST*_ values, Above diagonal = *P* values.

Bold values are significant, and asterisks represent significance values (*P < 0.05; **P < 0.01; ***P < 0.001).

N Nearshore O Offshore

**Figure 5:**
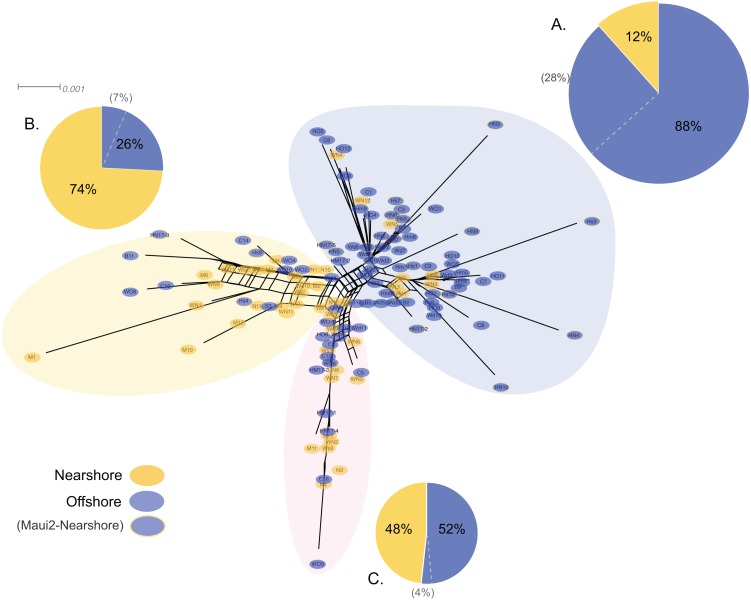
Diagrams of neighbor-net tree networks for Oahu and Maui *P. lobata* populations based on unphased H2 sequences. Genetic structure is stronger across human-impacted habitats than among islands in the coral *Porites lobata* NeighborNet phylogenetic networks generated by SplitsTree, color-coded by habitat-based genetic clusters: Blue represents the offshore group (including Maui2-nearshore population), and yellow represents the two genetically-close nearshore populations of Oahu and Maui1. The pie charts show the proportion of sequences present in each group. The gray numbers in () represent the proportion of Maui2 nearshore population.

Genetic structure of *P. lobata* across islands was also visualized using network analysis, which revealed three major clusters of H2 sequences, similar to the results from the Oahu populations (Fig. 5). Grouping by habitat-based genetic groups, Cluster 1 was dominated by the offshore type (including Maui2-nearshore) (88%), Cluster 2 was dominated by nearshore individuals (74%), and Cluster 3 had approximately same proportion of nearshore and offshore types, depicting separation of offshore and nearshore individuals, especially for Oahu and Maui1 populations. No clear pattern was observed based on geographic locations (Fig. S4).

## Discussion

Previous work on *P. lobata* at the Archipelagic scale (Polato et al., 2010) found IBD, although habitat-level variation was not examined. At a much smaller spatial scale considered here, with explicit sampling of contrasting habitats from across this strong environmental gradient, the pattern of genetic structure we observed for *P. lobata* in Hawaii did not follow IBD (Mantel Test, *r* =  − 0.091, *P* = 0.54). Instead, a pattern that resembles IBE was revealed, with a correlation between habitat types irrespective of geographic distance; the pairwise *F*_*ST*_ values revealed that offshore individuals from two separate islands (>100 km) were genetically closer to each other than to their geographically closest nearshore individuals (0.3–2 km), and nearshore individuals from two islands were also typically genetically closer to each other than to corals from adjacent offshore sites (Table 5, Fig. 5). Other studies that examined genetic differentiation on multiple scales show that patterns of IBD can be scale dependent. For example, Gorospe and Karl found distance (2013) and depth (2015) dependent patterns of coral genetic relatedness on a single patch reef, but this pattern was no longer detectable for analyses on an inter-reef scale (Gorospe & Karl, 2013). Furthermore, because of intra-reef patterns of genetic diversity and clonality, it is possible that sampling design may bias population-level patterns of genetic diversity (Gorospe, Donahue & Karl, 2015). However, we do not think this potential bias presents a problem in our system, since *P. lobata* is a broadcasting spawning species and shows little clonality (Boulay et al., 2012; Boulay et al., 2013; Schweinsberg, Tollrian & Lampert, 2016), particularly in Hawaii (Polato et al., 2010).

Similar patterns of small-scale genetic structure have been observed in several reef building corals (Barshis et al., 2010; Bongaerts et al., 2010; Carlon & Lippé, 2011). In the case of *Favia fragum*, the “Tall” ecomorph is a seagrass specialist with morphological adaptations to minimize sediment impacts, whereas the “Short” ecomorph shows morphological specializations for coral reef habitats that decrease its fitness in seagrass beds (Carlon & Budd, 2002). These traits are highly heritable and divergent selection appears to be driving reproductive isolation among these morphs and ongoing diversification in these incipient species (Carlon & Lippé, 2011). Here, we find a similar pattern of repeated genetic differentiation across strong ecological gradients driven by anthropogenic impacts (between adjacent sites 0.3–2 km apart), but little evidence of differentiation among similar habitats more than 100 km distant. Moreover, corals from a control site that lacks such a strong environmental gradient (Maui2) did not show the significant genetic structure documented at the other sites, which further suggests the potential role of environment in forming the observed genetic patterns (IBE).

Additionally, for the Oahu populations, differences in microskeletal morphology have been reported between the nearshore and offshore sites (Tisthammer & Richmond, 2018), similar to the correlation seen between genetic distance and microskeletal morphology across broader geographic regions in *P. lobata* (Forsman et al., 2015). The most noticeable difference was the height of pali (inner vertical skeletal structure that usually exists in a set of eight in *P. lobata*) within a corallite (the structure associated with individual polyps); nearshore corals had taller and more pronounced pali than the offshore ones. Because exact functions and heritability of the traits are unknown, whether the observed morphological differences are due to divergent selection cannot be answered at this point. However, the study suggested that the differences might be due to potential beneficial roles played by larger pali in shedding sediments in turbid water (Tisthammer & Richmond, 2018), similar to the case of the Caribbean coral *F. fragum* (Carlon & Budd, 2002; Carlon & Lippé, 2011; Carlon et al., 2011). Correlation between the morphological and genetic distances reported in the study is consistent with the idea of the likely role of environment in observed divergence.

Although we cannot rule out the possibility of prevailing oceanographic currents or barriers playing a role in producing our observed pattern of genetic patchiness, detailed studies on water movement suggests little dispersal barrier between the nearshore and offshore sites. In the bay at the Oahu site, surface currents primarily flow west due to the prevailing trade-winds (offshore to nearshore). The below surface current movement seems to be more complex, and is generally towards the east (nearshore to offshore) with the presence of small eddies, at least during the summer (Presto et al., 2012). Eddies would increase the larval retention time in the bay during the summer spawning season, especially for *Porites* species that produce neutrally buoyant gametes (Hunter, 1988). For Maui sites, the detailed water movement around the study areas revealed the presence of shear zones between the shallower (<15 m) nearshore and the deeper portions of the reef due to opposing flow directions during the coral reproductive season (summer), suggesting local retention of larvae (Storlazzi et al., 2006; Storlazzi & Field, 2008). While these shear zones could contribute as a gene flow barrier between the shallower and deeper areas, our offshore sites were located within the nearshore southward-flow dominated area, based on their depths and distances from the shore, thereby eliminating the probability of existence of strong oceanographic barriers between the nearshore and offshore study sites.

It is particularly interesting to find not only this pattern of clear genetic partitioning within a bay is repeated on a neighbor island, but also the genetic similarity exists between sites with similar histories of anthropogenic stress from the two separate islands, which points to the possibility of local processes acting to homogenize these geographically-separated sites. The nearshore sites at Oahu and Maui1 have experienced deteriorating water and substrate quality due to terrestrial runoff from urbanization of adjacent watersheds over the past century. This repeated pattern implies a possibility of similar underlying processes at both locations. We suspect that the environmental decline has likely limited new recruitment to the affected nearshore sites (Puritz & Toonen, 2011), and may have placed the populations under local selection, similar to what was seen in the Caribbean coral *F. fragum* (Carlon & Budd, 2002; Carlon & Lippé, 2011). The genetic markers used here are unlikely to be the direct targets of selection, and therefore, the observed genetic-environment association may result from linkage or be attributed to coupling of endogenous (intrinsic) and exogenous barriers, as theorized by Bierne et al. (2011). According to Bierne et al. (2011), intrinsic barriers that are independent of environment often play an important role in forming genetic-environmental associations through coupling with exogenous factors, rather than ecologically based divergent selection alone. Although our understanding on how selection affects population differentiation at neutral markers is limited at this point (Bierne et al., 2011), the coupling hypothesis may explain parallel genetic differentiation across independent putatively neutral markers observed in our study. Furthermore, there appears to be reduced genetic diversity in the nearshore habitats (Table 2, Table S1), which may represent a subset of the standing genetic variation of the larger population, thereby representing a limited number of individuals capable of surviving in the nearshore habitats. Regardless of the precise underlying mechanism, the role of environment in shaping the observed genetic divergence appears more important than geographic distance in this case.

Additional work is also needed to determine the specific environmental drivers likely to result in selection across these environmental gradients that generate the observed pattern. As discussed earlier, there are many factors, both natural and anthropogenic, that contrast between nearshore and offshore environments (e.g., salinity, irradiance, UV exposure, temperature, pH, wave exposure, nutrients, and biological community). Also, fine-scale water movements that the oceanographic-scale studies cannot capture (isolation by resistance, Thomas et al., 2015) may be contributing to physical barriers to reproduction. Any of these factors could contribute to create genetic partitioning between nearshore and offshore sites. For example, a comparable pattern of genetic structure has been observed across a particularly strong temperature gradient between the back-reef and forereef *P. lobata* populations in the areas with negligible terrestrial runoff or pollution in American Samoa (Barshis et al., 2010). However, in Hawaii we did not see such differentiation between nearshore and offshore sites at our less-impacted reference (Maui2), which raises a question of a potential role of other local abiotic or biotic factors affecting the observed pattern. It will be interesting to continue to observe whether this differentiation is transient and of little evolutionary importance, or whether it progresses towards incipient speciation, as it appears to have done in the Caribbean coral *F. fragum* (Carlon & Budd, 2002; Carlon & Lippé, 2011). Indeed, our finding may shed light on the common pattern of ‘chaotic genetic patchiness’ (Johnson & Black, 1982) so commonly reported among population genetic studies of marine organisms, in which geographically proximate populations show greater genetic differentiation than those from distant sites (e.g., Johnson & Black, 1984; Hogan, Thiessen & Heath, 2010; Toonen & Grosberg, 2011; Eldon et al., 2016).

The ecological diversification of reef building corals over a small spatial scale, despite ongoing gene flow, also provides a rare example of genetic divergence in the absence of spatial barriers to gene flow, indicating that divergent natural selection can act as an evolutionary driver of reproductive isolation (Carlon & Budd, 2002; Bongaerts et al., 2010; Bongaerts et al., 2011; Carlon et al., 2011). Our results may extend another example of *P. lobata* in Hawaii to these findings, which showed potential occurrence of similar diversification process across steep environmental gradients driven. This may represent the initial stages of adaptive diversification, as seen in other marine species from the Hawaiian Archipelago (e.g., limpets, Bird et al., 2011; Bird, 2011). There is clearly some genetic connectivity among adjacent islands, congruent to previous studies (Polato et al., 2010), and hence the observed differentiation across these steep ecological gradients in spite of high dispersal potential (Storlazzi & Field, 2008; Presto et al., 2012) may be the early phases of speciation with gene flow (Feder, Egan & Nosil, 2012). Whether this initial stage of divergence among habitats is transient or has the potential to progress to later stages remains to be seen, but our results and others (Bird et al., 2011; Bird, 2011) indicate that this initial stage can be realized even in a broadcasting species with high dispersal potential. Together, these results add to the growing evidence that the initial phase of speciation is possible without geographic isolation, and lend support to the hypothesis that ecological speciation (*sensu* Bowen et al., 2013) may be more common in the sea than believed previously.

Finally, the potential reduction in intraspecific genotypic diversity within the populations of corals under higher levels of environmental stressors can be a warning for the future of reefs. Focusing on the usual metrics of coral reef health and resilience, specifically coral cover and species diversity, misses the very concerning loss of genotypic diversity essential to population resilience in a changing world. The importance of molecular data is apparent in tracking the invisible changes that can lead to local extinctions, and hence, such tools need to be further refined and more broadly applied to the helping ensure the persistence of coral reefs as a legacy for the future.

## Conclusions

Our results show that the broadly distributed broadcast spawning coral *P. lobata* exhibits very fine-scale (0.3∼2 km) genetic structure, and environmental drivers across habitat types appear to have a stronger effect in forming such genetic structure (IBE) than geographic distances (IBD) in coastal areas that are heavily affected by anthropogenic stressors. Genetic similarity found between Oahu and Maui1 nearshore populations suggest that the observed genetic structure may be driven by similar underlying evolutionary mechanisms, including adaptation to environmentally driven factors. Our results also highlight the importance of thorough sampling among habitats at small scales; we could easily overlook such important local genetic differences, and may mistakenly conclude that populations are uniform across the landscape without thorough sampling. Although our samples are from limited locations, these results demonstrate that understanding small-scale genetic variation and diversity can provide important information on the ecological basis of genetic diversity and differentiation, which must be understood to effectively implement future coral reef conservation efforts.

##  Supplemental Information

10.7717/peerj.8550/supp-1 Figure S1 Close-up map of sampling locations at Maunalua Bay, Oahu, Hawaii Nearshore site is located at the mouth of the embayment around which the urban population center of Hawaii Kai has developed. This area sees intense human use and a strong anthropogenic impact gradient to Offshore site at uninhabited Koko Head. Though the offshore site locates relatively close to the shore, its conditions are closer to those of offshore due to the unique topography (Map provided by Curt Storlazzi, U.S. Geological Survey and used with permission).

10.7717/peerj.8550/supp-2 Figure S2 Close-up map of sampling locations at Hanakao’o Beach Park, Wahikuli, West Maui, Hawaii Nearshore site is located close to the beach where run-off and human activities are concentrated. The run-off and the unique current patterns create drastic differences in water quality between the nearshore and offshore sites (Map services and data available from U.S. Geological Survey, National Geospatial Program).

10.7717/peerj.8550/supp-3 Figure S3 Morphological identification of *Porites* species corals used in this study Close-up images of polyps and corallites of *Porites lobata* (A, B) and *Porites evermanni* (C, D). Live polyps (A, C) and bleached corallite skeleton (B, D) of the two species. Images taken under a stereo microscope, highlighting the morphological differences to distinguish between *P. evermanni* (C, D) which has a smoother colony surface, whereas corallites of *P. lobata* (A,B) are contoured and deeper.

10.7717/peerj.8550/supp-4 Figure S4 Allele networks for all *Porites lobata* sampled from the islands of Oahu and Maui NeighborNet phylogenetic networks generated by SplitsTree, color-coded by geographic region as shown in Fig. 1. Pie charts represent the proportion of sequences within each cluster.

10.7717/peerj.8550/supp-5 Table S1 Genetic diversity comparison between the nearshore (N) and offshore (O) *P. lobata* populations from Oahu (Maunalua Bay) based on 17,850 single nucleotide polymorphic loci The number of heterozygous sites per individual was obtained using VCFtools, and the total number of polymorphic loci was obtained using Arlequin (for the nearshore population, showing the average over two individuals).

10.7717/peerj.8550/supp-6 Table S2 Analysis of Molecular Variance (AMOVA) results for *P. lobata* AMOVA tables for (A) Individuals by geographic sampling sites, (B) Individuals sampled within habitat types across islands (nearshore vs offshore individuals from Oahu and Maui1).

10.7717/peerj.8550/supp-7 File S1 Characteristics of Genetic Markers

## Additional Information and Declarations

### Field Study Permissions

The following information was supplied relating to field study approvals (i.e., approving body and any reference numbers): Coral samples were collected under the State of Hawaii Division of Aquatic Resources, Special Activity Permits #2013-26, 2014-64, 2015-06, and 2017-16.

### Data Availability

The following information was supplied regarding data availability: All raw sequence data are available at GenBank: ITS: KY493091–KY493160; H2: KY502280–KY502376 and MF629151–MF629152; CR: KY502373, KY502374 and KY502375. The phased H2 sequences used in the analysis are available on Figshare: Toonen, Rob (2019): Phased_H2_sequence_data.docx. figshare. Dataset. https://doi.org/10.6084/m9.figshare.10315430.v1.
