# Supplementary figures and images for: Genetic structure is stronger across human-impacted habitats than among islands in the coral Porites lobata
Source: PeerJ. 2020 Feb 18;8:e8550. doi: 10.7717/peerj.8550 (PMC7034377; doi:10.7717/peerj.8550)

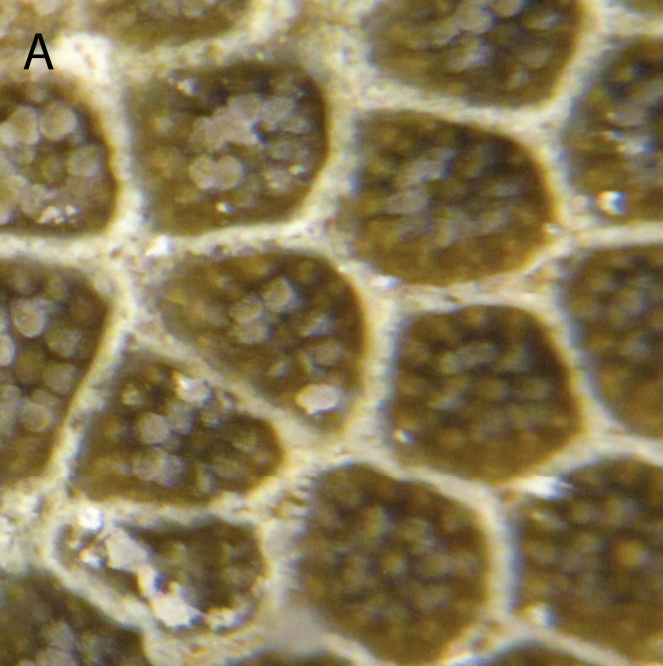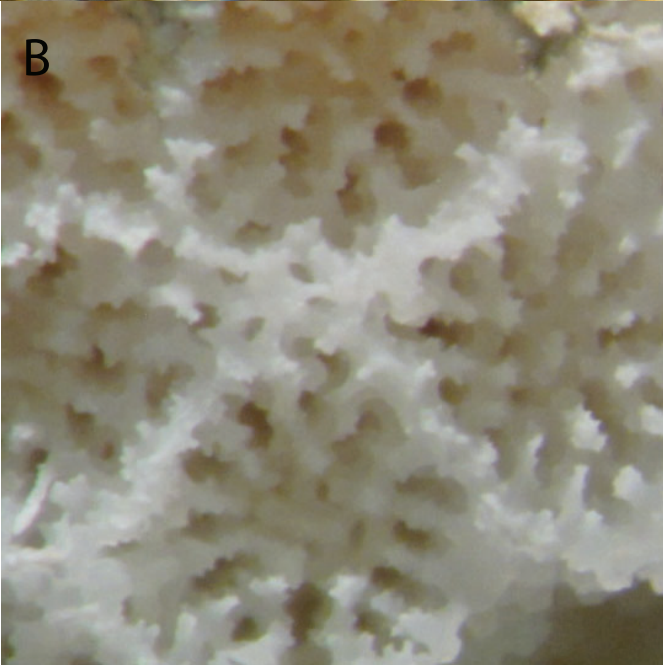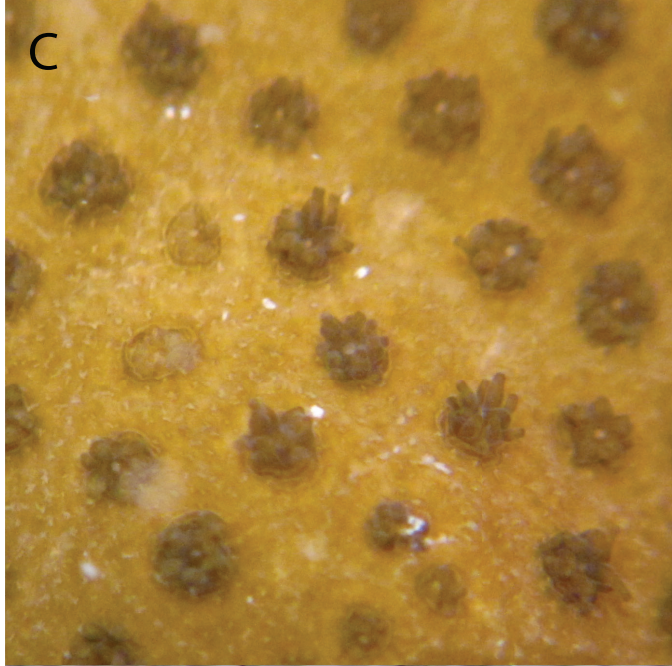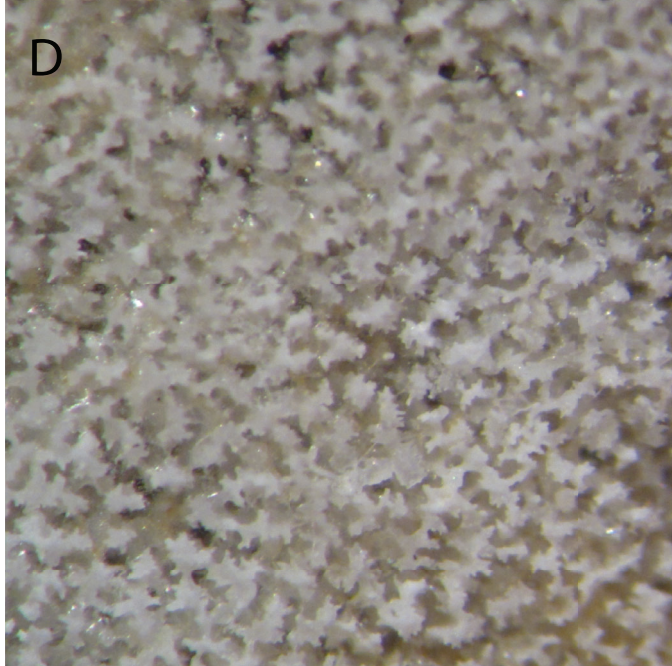

Supplement: Figure S3 — Close-up images of polyps and corallites of Porites lobata (A, B) and Porites evermanni (C, D). Live polyps (A, C) and bleached corallite skeleton (B, D) of the two species. Images taken under a stereo microscope, highlighting the morphological differences to distinguish between P. evermanni (C, D) which has a smoother colony surface, whereas corallites of P. lobata (A,B) are contoured and deeper. [file peerj-08-8550-s003.pdf]
