# Supplementary material for: Genetic structure is stronger across human-impacted habitats than among islands in the coral Porites lobata
Source: PeerJ. 2020 Feb 18;8:e8550. doi: 10.7717/peerj.8550 (PMC7034377; doi:10.7717/peerj.8550)
Supplement: Table S1 — The number of heterozygous sites per individual was obtained using VCFtools, and the total number of polymorphic loci was obtained using Arlequin (for the nearshore population, showing the average over two individuals). [file peerj-08-8550-s005.docx]

**Supplemental Table 1: Genetic diversity comparison between the nearshore and offshore *P. lobata* populations from Oʻahu (Maunalua Bay) based on 17,850 single nucleotide polymorphic loci.** The number of heterozygous sites per individual was obtained using VCFt
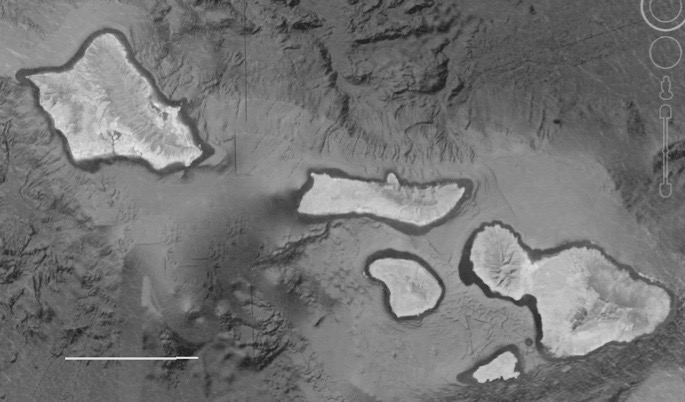
ools, and the total number of polymorphic loci was obtained using Arlequin (for the nearshore population, showing the average over two individuals).

|  | SampleID | Heterozygous sites (HET)^1^ | Ave. no. of polymorphic loci across 2 individuals^2^ |
| --- | --- | --- | --- |
| Offshore  (O) | Coral1 (C6) | 7621 |  |
|  | Coral2 (C16) | 7518 |  |
|  | Average | **7569.5** | **11105** |
| Nearshore  (N) | Coral6 (N1) | 7267 |  |
|  | Coral7 (N3) | 6631 |  |
|  | Coral8 (M2) | 7200 |  |
|  | Coral9 (M7) | 7345 |  |
|  | Coral10 (M12) | 7622 |  |
|  | Average | **7213.0** | **10255.4 ± 43.3** |
|  |  |  |  |

^1^ t-test: t = -2.0942, df = 4.6575, p-value = 0.09451

^2^ no statistical test since n=1 for Offshore

**Supplemental Table 2: AMOVA results of *P. lobata.*** (A) Populations across islands, (B) populations across habitat types; nearshore vs offshore individuals from Oʻahu and Maui1 (B).

| A | Source of Variation | Variance components | % Variance | Fixation indices |  |
| --- | --- | --- | --- | --- | --- |
|  | Among groups (islands) | 0.00135 | 0.03 | F_CT_ = 0.0003 |  |
|  | Among populations within groups | 0.24897 | 6.35 | F_SC_ = **0.063**^***^ |  |
|  | Among individuals Within populations | 1.51485 | 38.62 | F_IS_ = **0.41**^***^ |  |
|  | Within individuals | 2.15769 | 55.00 | F_IT_ = **0.45**^***^ |  |

| B | Source of Variation | Variance components | % Variance | F_ST_ |  |
| --- | --- | --- | --- | --- | --- |
|  | Between sites | 0.2360 | 5.96 | **0.060^***^** |  |
|  | Within sites | 1.3906 | 35.12 |  |  |
|  | Within individuals | 2.3333 | 58.92 |  |  |
