# Supplementary material for: Genetic structure is stronger across human-impacted habitats than among islands in the coral Porites lobata
Source: PeerJ. 2020 Feb 18;8:e8550. doi: 10.7717/peerj.8550 (PMC7034377; doi:10.7717/peerj.8550)
Supplement: Table S2 — AMOVA tables for (A) Individuals by geographic sampling sites, (B) Individuals sampled within habitat types across islands (nearshore vs offshore individuals from Oahu and Maui1). [file peerj-08-8550-s006.docx]

| A | Source of Variation | Variance components | % Variance | Fixation indices |  |
| --- | --- | --- | --- | --- | --- |
|  | Among islands | 0.00135 | 0.03 | F_CT_ = 0.0003 |  |
|  | Among populations within islands | 0.24897 | 6.35 | F_SC_ = **0.063**^***^ |  |
|  | Among individuals within populations | 1.51485 | 38.62 | F_IS_ = **0.41**^***^ |  |
|  | Within individuals | 2.15769 | 55.00 |  |  |

| B | Source of Variation | Variance components | % Variance | F_CT_ |  |
| --- | --- | --- | --- | --- | --- |
|  | Among sites | 0.2360 | 5.96 | **0.060^***^** |  |
|  | Between habitat types | 1.3906 | 35.12 |  |  |
|  | Within individuals | 2.3333 | 58.92 |  |  |
