# Supplementary material for: Genetic structure is stronger across human-impacted habitats than among islands in the coral Porites lobata
Source: PeerJ. 2020 Feb 18;8:e8550. doi: 10.7717/peerj.8550 (PMC7034377; doi:10.7717/peerj.8550)
Supplement: File S1 [file peerj-08-8550-s007.pdf]

Characteristics of Genetic Markers

Comparisons among the three types of genetic markers revealed that the non-coding nuclear marker ITS had the highest levels of genetic variability relative to the coding nuclear region H2 and the mitochondrial marker CR. The level of polymorphism in ITS was particularly high: 77 polymorphic sites were observed across the 707 bp (10.9%) including indels. Seventy six polymorphic sites across the1352 bp (5.6%) were observed in H2, and in CR only two sites were polymorphic across the 366 bp (0.55%). The total number of indels observed in ITS was 50, while H2 and CR did not contain any indels. In the ITS marker, there were four major indels with base pairs that were two or more, and the longest indel observed was 23 bp. Polymorphic sites were observed scattered throughout the maker length in both H2 and ITS (Fig. 2). In ITS, 33% of polymorphic sites were present in only one or two alleles, while in H2, 63% of polymorphic sites were present in one or two alleles. These results were reflected in a mean gene diversity of polymorphic sites that was more than twice the value in ITS (0.310 ± 0.192) compared to the value in H2 (0.140 ± 0.167).

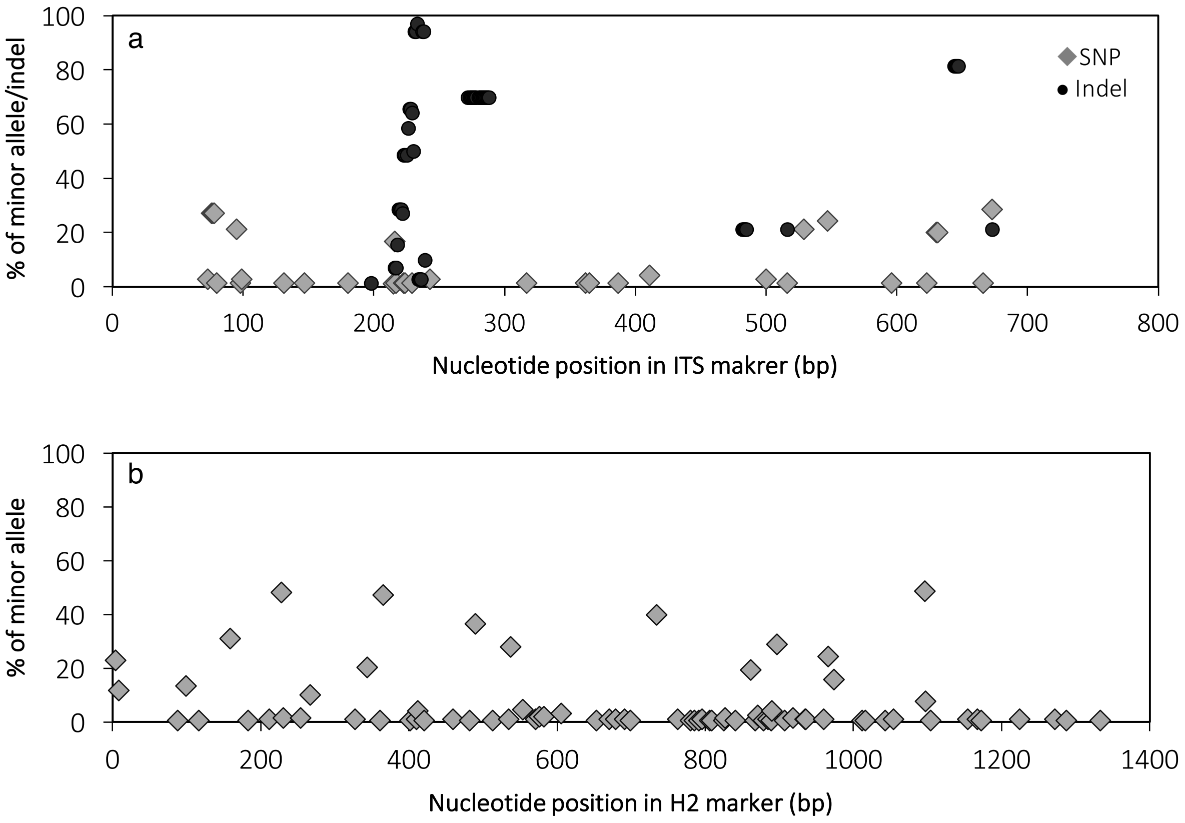

Locations of polymorphic sites across the genetic markers and their frequencies: (a) ITS and (b) Histone2 markers.
